# Allelic Variations at Four Major Maturity *E* Genes and Transcriptional Abundance of the *E1* Gene Are Associated with Flowering Time and Maturity of Soybean Cultivars

**DOI:** 10.1371/journal.pone.0097636

**Published:** 2014-05-15

**Authors:** Hong Zhai, Shixiang Lü, Yueqiang Wang, Xin Chen, Haixiang Ren, Jiayin Yang, Wen Cheng, Chunmei Zong, Heping Gu, Hongmei Qiu, Hongyan Wu, Xingzheng Zhang, Tingting Cui, Zhengjun Xia

**Affiliations:** 1 Key Laboratory of Soybean Molecular Design Breeding, Northeast Institute of Geography and Agroecology, Chinese Academy of Sciences, Harbin, China; 2 University of Chinese Academy of Sciences, Beijing, China; 3 Soybean Research Institute, Jilin Academy of Agricultural Sciences, Changchun, China; 4 Jiangsu Academy of Agricultural Sciences, Nanjing, China; 5 Mudanjiang Branch of Heilongjiang Academy of Agricultural Sciences, Mudanjiang, China; 6 Huaiyin Institute of Agricultural Sciences of Xuhuai Region in Jiangsu, Huaian, China; 7 College of Life Sciences, Shandong Normal University, Jinan, China; University of Illinois, United States of America

## Abstract

The time to flowering and maturity are ecologically and agronomically important traits for soybean landrace and cultivar adaptation. As a typical short-day crop, long day conditions in the high-latitude regions require soybean cultivars with photoperiod insensitivity that can mature before frost. Although the molecular basis of four major *E* loci (*E1* to *E4*) have been deciphered, it is not quite clear whether, or to what degree, genetic variation and the expression level of the four *E* genes are associated with the time to flowering and maturity of soybean cultivars. In this study, we genotyped 180 cultivars at *E1* to *E4* genes, meanwhile, the time to flowering and maturity of those cultivars were investigated at six geographic locations in China from 2011 to 2012 and further confirmed in 2013. The percentages of recessive alleles at *E1*, *E2*, *E3* and *E4* loci were 38.34%, 84.45%, 36.33%, and 7.20%, respectively. Statistical analysis showed that allelic variations at each of four loci had a significant effect on flowering time as well as maturity. We classified the 180 cultivars into eight genotypic groups based on allelic variations of the four major *E* loci. The genetic group of e1-nf representing dysfunctional alleles at the *E1* locus flowered earliest in all the geographic locations. In contrast, cultivars in the E1E2E3E4 group originated from the southern areas flowered very late or did not flower before frost at high latitude locations. The transcriptional abundance of functional *E1* gene was significantly associated with flowering time. However, the ranges of time to flowering and maturity were quite large within some genotypic groups, implying the presence of some other unknown genetic factors that are involved in control of flowering time or maturity. Known genes (e.g. *E3* and *E4*) and other unknown factors may function, at least partially, through regulation of the expression of the *E1* gene.

## Introduction

It is generally considered that soybean has been domesticated in China for several thousand years [1]. During the domestication and breeding processes, the time to flowering and maturity underwent natural and human selections since these traits affect geographic distribution, sowing time and final yield of soybean cultivars. Latitudinal distribution for a given cultivar is typically restricted to a limited north south zone for the maximal yield. Since soybean is a short-day crop, soybean cultivar acquired a certain photoperiodic insensitivity to adapt to higher latitude regions where there is a longer daylength in the summer and a shorter frost-free period a year. A maturity group (MG 000, MG 00, MG 0, MG I ∼ MG X) system has been well developed to estimate the range of adaptability to latitudinal or geographic zones of soybean cultivars in the USA and Canada [2], [3]. MG 000, MG 00, and MG 0 are the earliest maturity cultivars and are mainly distributed in production areas of the southern Canada [4], while MG I and MG II are typically grown in the northern region of the USA, the rest of the groups are succeedingly grown further south [5]. Although this system has been practically used for several decades in soybean breeding and soybean production, assignment of a cultivar into a maturity group is time-consuming, and is sometimes hard due to the ambiguous phenotypic data [5].

As early as 1920s, researchers began to study the genetic factors regulating flowering time [6]. *E1* to *E8* loci, known as the *E* series, have been genetically identified (*E1* and *E2* [7], *E3* [8], *E4* [9], *E5* [10], *E6* [11], *E7* [4], and *E8* [12]). Additionally, the *J* locus for long juvenile was also genetically detected [13]. The *E* series of loci also underlie the maturity or the duration of the reproductive phase (DRP) in soybean [14]. Relationship between maturity groups and genotypes of the *E* loci was inferred by genetic study using Harosoy or Clark near isogeneic lines (NILs) [5].

Recently, the availability of soybean genomic information has accelerated positional cloning in soybean [15]. To date, the molecular bases for *E1* to *E4* loci have been uncovered [16]–[19], although other loci *e.g. E5* to *E8* loci still remain unknown. Genetic analysis indicated the *E3* gene is partially dominant over *E4* [20], and both genes respond to the quality of light [20], [21]. Recent progress made out that the *E3* and *E4* genes encode two phytochromes, GmPhyA3 and GmPhyA2 [16]–[17]. In *Arabidopsis*, the *GIGANTEA* (*GI*) gene plays an important role in the GI-CO-FT photoperiodic flowering pathway and also can directly regulate the *FT* gene [22], [23]. Positional cloning revealed that *GmGIa*, an ortholog of *GI* gene is responsible for the *E2* locus in soybean [18]. The *E2* locus is reported to have no strong association with photoperiodic response, and functional mechanism of the *E2* gene in soybean is still needed to be elucidated further [18].

Generally, the *E1* locus has major impact on flowering and maturity. The notice of its effect on flowering could trace back as early as 1920s when photoperiodism was discovered [24]. Although the *E1* was named in 1971 [7], the cloning of this gene was rather difficult since this locus is located in the pericentromeric region [25]. Xia and co-researchers successfully disclosed the molecular identity for the *E1* locus through nearly 10 years of fine mapping and functional confirmation [19]. The *E1* gene has a bipartite nuclear localization signal and a domain distantly related to the AP2 domain. Functional analysis showed nonfunctional *E1* alleles displayed an early flowering time phenotype, regardless of the genetic background at other *E* loci or daylength condition [19]. The strongly suppressed expression in short day condition also infers that this gene is strongly related to photoperiodic response as a flowering repressor, which is consistent with the results obtained in previous genetic studies[19]. In addition, the *E1* locus might have pleiotropic effects on other important agronomic traits [26], e.g. branching [27] and chilling tolerance [28].

Recently, Tsubokura et al. genotyped 63 accessions at the *E1*, *E2*, *E3* and *E4* genes using DNA markers, and also sequenced the promoter and coding regions of those genes to detect the genetic variation at single nucleotide level for 39 accessions [29]. The result showed that these allelic variations could explain about 62 to 66% of the phenotypic variation of flowering time among the 63 plant accessions used [29]. Xu et al (2013) studied 53 photoperiod insensitive soybean accessions including some cultivars from Heilongjiang, the most northern province in China, and classified them into 6 genotypic groups using genotype data of *E1* to *E4* [30]. However, the general information on genotypic variations at the four known *E* loci among cultivars from different soybean growing areas or geographic regions with different range of photoperiod sensitivity in China is still lacking. Therefore, the aims of this study were to investigate the extent to which the genetic variation at *E1* through *E4*, as well as expression of the *E1* gene could explain the phenotypic variation of the time to flowering and maturity for cultivars collected from different soybean growing areas in China. The results obtained in this study will be useful for identification and cloning of new genes involved in flowering time or maturity, as well as for marker assisted selection in soybean breeding.

## Materials and Methods

### Soybean Cultivars and Accessions

A total of 180 cultivars were mainly obtained from the Gene Resource Center of Jilin Academy of Agricultural Sciences, China. The origin and other traits for these cultivars are listed in Table S1 in detail.

### Genotyping

A standard CTAB DNA extraction protocol was followed [31]. The genotyping of all cultivars at the *E1*, *E2*, *E3* and *E4* genes was performed using known DNA markers (Table S2). Electrophoresis was conducted by either agarose gel or high-efficiency genome scanning (HEGS) with non-denaturing 11–13% polyacrylamide separating gels and 5% stacking gels [32], [33]. The gels were stained with GelStain (Transgen, Beijing, China), and visualized with Gel Doc XR Molecular Imager System (Bio-Rad, USA).

#### (1) Genotyping of the *E1* Gene

**Figure 1:**
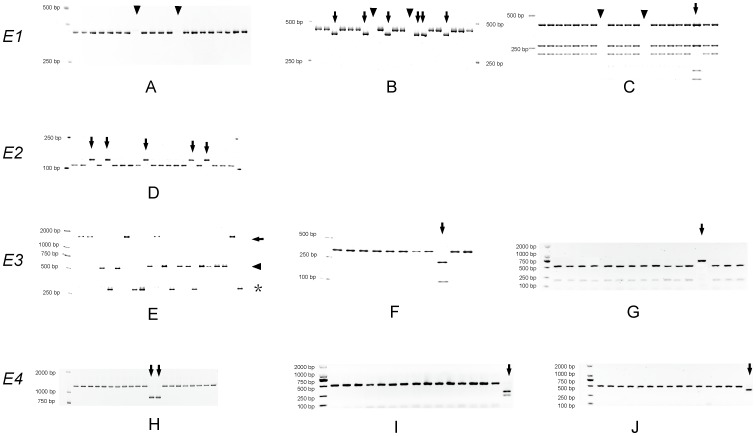
Genotyping methods for *E1* to *E4* loci. A–C: Genotyping of the *E1* gene. Fragment was amplified by primer pair of TI-Fw and TI-Rv (A), subsequently subjected to *Taq*I digestion (B) and *Hinf*I (C). The triangle represents the *e1-nl* type (lacking of fragment). The arrows in B and in C are representing *e1-as* and *e1-fs* genotypes, respectively. D: Genotyping of the *E2* gene, fragment was amplified with primer pair of SoyGI_dCAP_Dra_fw and SoyGI_dCAP_Dra_rv, after digestion of *Dra*I, the *e2* was cut into two fragments, while *E2* genotype remained uncut (arrows). E–G: Genotyping of the *E3* gene. E: bands with three sizes were generated using the mixed primers. Band specific for *E3-Mi* and *e3-tr* were indicated by arrow and star, respectively. A 557 bp fragment (triangle) could be amplified either for *E3-Ha* or *e3-Mo* genotypes. F: PCR product yielded from primer pair *E3*_08094FW and *E3*_08417RV were digested with *Mse*I. The *E3-Ha* genotype remained uncut, while *e3-Mo* allele (arrow) could be cut into 223 bp and 101 bp. G: specific determination of *E3-fs* type (arrow). CAPE primer pair *E3*-fsFW/*E3*-fsRV, restriction enzyme: *Ale*I. H-J: Genotyping of the *E4* gene. H: Using three mixed primers, a 837 fragment was specifically amplified from the *E4* allele (arrow). I: CAPE primer pair *e4-kam* specific for *e4-kam* gene (arrow), enzyme *Afl*II. J: CAPE primer pair *e4-kes* specific for *e4-kes* genotype (arrow), enzyme *Bsp*HI.

Genomic DNA was amplified with primer pair of TI-Fw and TI-Rv [19]. The PCR was performed using the following program: 30 cycles at 94°C for 20 s, 58°C for 30 s, and 72°C for 30 s. A 443/444 bp fragment (Figure 1A) was amplified and subjected to *Taq*I or *Hinf*I digestion. The banding patterns were used to distinguish among *E1*, *e1-as*, and *e1-fs* [19]. The PCR product amplified from *e1-as* allele was cut into two fragments, 410 bp and 34 bp by *Taq*I (arrows in Figure 1B,) while that amplified from *E1* or *e1-fs* allele remained uncut. For discrimination between *E1* and *e1-fs* alleles, the fragment amplified from *e1-fs* allele could be cut into four fragments (234,117,47 and 34 bp), while only three fragments were present for the *E1* allele. Besides, the *e1-nl* allele lacking the 443 bp band was indicated by triangles in Figure 1A to 1C.

#### (2) Genotyping of the *E2* Gene

The 142 bp fragment was amplified with primer pair SoyGI_dCAP_Dra_fw and SoyGI_dCAP_Dra_rv [18], [29] and subjected to *Dra*I digestion. The fragment amplified from *E2* allele remained uncut, while that from *e2* allele could be cut into 115 bp and 27 bp fragments (Figure 1D).

#### (3) Genotyping of the *E3* Gene

Genomic DNA was amplified with a primer set consisting of four primers, E3_08557FW, E3_09908RV, E3Ha_1000RV and E3T_0716RV [29]. A 1406 bp fragment could be specifically amplified from *E3-Mi* allele, while a 269 bp fragment could be specifically amplified from *e3-tr* allele. A 557 bp fragment could be amplified from either *E3-Ha* or *e3-Mo* alleles (Triangle, Figure 1E). For further discrimination between *E3-Ha* and *e3-Mo*, the PCR product was subsequently amplified with a primer pair of E3_08094FW and E3_08417RV, and subjected to *Mse*I digestion (Figure 1F). The fragment amplified from the *E3-Ha* allele remained uncut; while that from *e3-Mo* allele could be cut into 223 bp and 101 bp fragments. For specifically identification of *e3-fs* allele, the fragment (758 bp) was amplified with primer pair of e3-fsFW and e3-fsRV and subjected to *Ale*I digestion. The fragment amplified from the *e3-fs* allele remained uncut, while that amplified from the *E3-Ha* was cut into 552 bp and 206 bp fragments (arrows in Figure 1G).

#### (4) Genotyping of the *E4* Gene

Using a set of primers, PhyA2-For, PhyA2-Rev/E4 and PhyA2-Rev/E4 [29], an 837 bp fragment could be specifically amplified from the *e4-SORE1* allele, while a 1,229 bp fragment could be generated from other types of *E4* alleles (Figure 1H). For discrimination between *e4-kam*, *e4-kes* and the *E4* alleles, the PCR product was amplified with primer pair of e4-kamFW and e4-kamRV, and was digested with *Afl*II or *BspH*I. The fragment from *e4-kam* allele could be cut into 286 and 208 bp fragments by *Afl*II (Figure 1I). Also the fragments amplified from *e4-kes* allele could be cut into 399 and 95 bp by *BspH*I (Figure 1J). While the fragment from the *E4* allele remained uncut by either *Afl*II or *BspH*I.

### Phenotypic Observation

R1 to R8, the reproductive stages of soybean, were defined according to Fehr's system [34]. R1 refers the beginning of bloom when the opening of the first flower was found at any node on the main stem. R7 stands for the beginning of maturity when one normal pod on the main stem has reached its mature pod color (normally brown or tan); R8 refers full maturity when 95 percent of the pods have reached their mature pod color. For a given cultivar, each specific R stage is defined only when at least 50% of individual plants reached that stage. At least 15 plants for each cultivar per geographic location were grown for the phenotypic evaluation. For convenience, the data for R1, R7 or R8 of a given cultivar presented in a format of means ± standard derivation (s.d.) in this study were recorded as the number of days after emergence to the stage of R1, R7 and R8, respectively.

**Figure 2:**
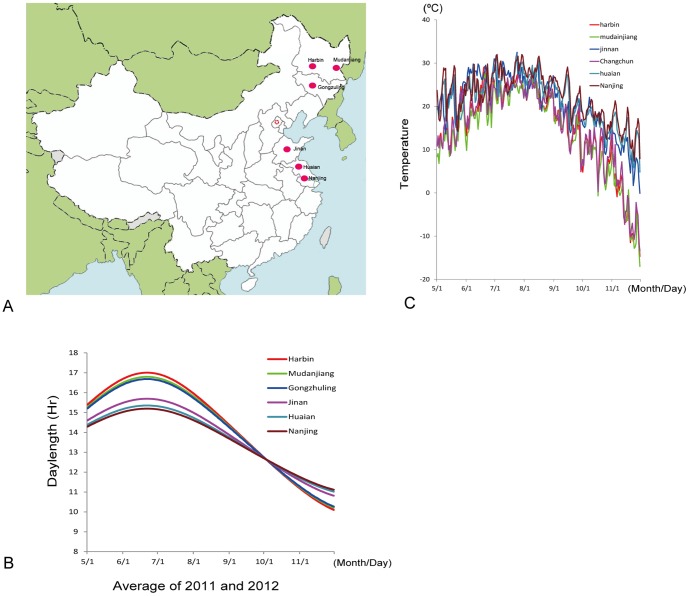
Geographic locations, daylength, and temperature of six experimental sites. A: The geographic locations of the six experimental sites. B: the average day length (hr) between 2011 and 2012. C: The changes in temperature recorded in 2011. Since there was no temperature data available in Gongzhuling (43°53′ N, 124°84′E), we used the data from the neighboring city Changchun (43°88′ N, 125°35′ E) (60 Km apart) instead.

Six geographic locations were chosen for evaluating photoperiodic responses. Three of them were located within Northern latitudes of 43° to 46°N, the critical regions for photoperiod response since most cultivars can mature during the frost free period and display contrasting differences in time to flowering. The other three different latitudinal locations were located successively in southern area (Figure 2A). Six locations are: 1 Harbin (HRB): Research field at the Campus of Northeast Institute of Geography and Agroecology, Harbin, Heilongjiang (45°70′ N, 126°64′ E); 2. Mudanjiang (MDJ): Mudanjiang Research Station, Heilongjiang Academy of Agricultural Science (44°42′N, 129°52′E); 3. Gongzhuling (GZL): Gongzhuling Research Station, Jilin Academy of Agricultural Science, Gongzhuling, Jilin (43°53′ N, 124°84′ E); 4. Jinan (JN): Campus of Shandong Normal University, Jinan, Shandong (36°66′ N, 117°17′ E); 5. Huaian (HA): Huaiyin Research Station, Jiangsu Academy of Agricultural Science, Huaian, Jiangsu (33°57′ N, 119°04′ E); 6. Nanjing (NJ): Luhe Research Station, Jiangsu Academy of Agricultural Science, Nanjing, Jiangsu (32°31′ N, 118°82′ E) (Figure 2A). We did not include locations further north than Harbin (45°70′ N, 126°64′ E), considering the most cultivars collected from the South might not reach flowering (R1) before frost. Also we did not include any location further south than Nanjing (32°31′ N, 118°82′ E), since flowering time or maturity time in the short-day condition become shorter and similar for the majority of cultivars collected. Based on the first two years' (2011 and 2012) results (Table S3), around 50 representative cultivars mainly showing contrasting R1 or R7 or R8 within the same genetic group (see “Results” section) were selected for further phenotypic confirmation in 2013.

### Quantitative Real-time PCR

Quantitative real-time PCR (qRT-PCR) analysis was performed for plant materials taken from the location of Harbin on May 20, 2012 when the day length was 16.10 hr. The cultivars were performed for qRT-PCR at this location were star (*) marked in the accession column in Table S1. The upmost fully expanded leaves from the apical meristem were sampled from 2.5 to 3 hr after dawn, 14 days after emergence. Total RNA was extracted using TRIzol (Life Technologies) method. The isolated RNA was then subjected to reverse transcription using the SuperScript III Reverse Transcriptase kit. Quantitative real-time PCR was performed on each cDNA sample with the SYBR Green Master Mix (TransStart Top Green qPCR SuperMix) on Bio-Rad Chromo4 Detection System according to the manufacturer's protocol. The measured Ct values were converted to relative copy-numbers using the ΔΔCt method. Amplification of *TUA5* (*Glyme05g29000.1*) was used as an internal control to normalize all data. *E1* expression level of Kariyutaka was used as a reference. Primers used were *TUA5*-F 5′-TGCCACCATCAAGACTAAGAGG and *TUA5*-R 5′-CTCTAATGGCGGCATCAAG; *E1*-F 5′–CACTCAAATTAAGCCCTTTCA and *E1*-R 5′- TTCATCTCCTCTTCATTTTTGTTG; Three fully independent biological replicates were obtained and subjected to real-time PCR run in triplicate. Raw data were standardized as described previously [35].

### Weather Data Collection and Statistical Analysis

The temperature data were downloaded from the National Meteorological Information Center (http://cdc.cma.gov.cn. Accessed 2014, March 30). The daylength data were calculated at time.ac.cn/calendar/calendar.htm for the six sites where we performed the phenotypic observation. In order to statistically evaluate the effects of allelic variation for each *E* locus and their combinations on flowering time and maturity, multivariate analysis was performed using IBM SPSS Statistics 17.0, www-01.ibm.com/software/analytics/spss, based on generalized linear models. The Type III Sum of Squares was used to test effects between subjects. All other statistical analysis, e.g. comparison between different genetic groups, was performed using GraphPad Prism Version 5.0 for Windows, GraphPad Software (San Diego California USA, www.graphpad.com).

## Results

### 1. Correlation of Time to Flowering and Maturity among Different Geographic Locations

After careful selection, 180 cultivars were used in this study, most of which were elite germplasm lines or cultivars extensively used in China, previously or currently. Also, cultivars from the USA, Japan, and European were included for comparison. Several cultivars carrying a specific genotype, e.g. Sakamotowase for *e1-fs*, Kariyutaka for *E1e3e4* were also included as control for monitoring the performance in genotyping and phenotypic evaluation. The phenotypic evaluation for the time to flowering and to maturity, as well as the other agronomic traits was conducted in the six locations from 2011 to 2012 (Figure 2, Table S1).

Daylength during growth season at different sites was variable as shown in Figure 2. Considering the frost-free period and temperature, we generally sowed seeds as the local farmers did from late April to the middle of May for the northern sites: Harbin, Mudanjiang and Gongzhuling, or in June for the three southern sites, Jinan, Huaian, and Nanjing. The number of days from sowing to emergence was depending on temperature and soil moisture. For individual plant, R1 was recorded as the number of days from emergence to first flowering open. At the population level of a cultivar, R1 was recorded when more than 50% percentage of individual plants reached the R1 stage. Some data for R1 or R8 were missing mainly because cultivars did not reach the R1 stage before frost or died from diseases during germination. The cultivars originally from southern regions, e.g. nearby the Yangtze River, flowered late in the three northern sites, Harbin, Gongzhuling and Mudanjiang.

**Figure 3:**
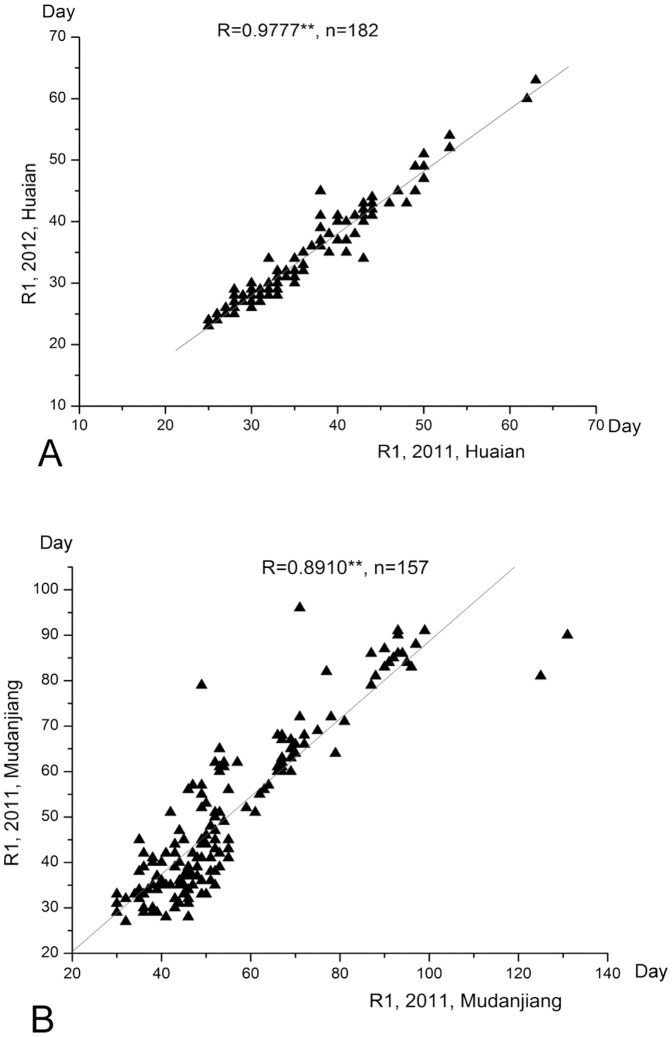
The correlation analyses of the time to flowering (R1) between 2011 and 2012. A: Huaian; B: Mudanjiang.

Generally, R1 is a relatively stable ecological character for a cultivar as indicated by higher correlation coefficients between different sites. At the southern sites of Huaian and Nanjing, the R1 of 2011 and 2012 were significantly correlated with correlation coefficient of 0.977** (hereinafter ** stands P<0.01) and 0.938** (Table 1, Figure 3). However, for the other sites, the correlation coefficients ranged from 0.790** to 0.923** (Table 1, Figure 3), the fluctuations in correlation coefficients were possibly due to the influence of fluctuated environmental factors e.g. soil moisture, temperature from sowing to emergence between different years or between different sites.

**Table 1:** The correlation matrix for flowering time (R1) between all possible pairs at six locations in 2011 and 2012.

	Harbin, 2011	Harbin, 2012	Mudanjiang, 2011	Mudanjiang, 2012	Gongzhuling, 2011	Gongzhuling, 2012	Jinan, 2011	Jinan, 2012	Huaian, 2011	Huaian, 2012	Nanjing, 2011	Nanjing, 2012
**Harbin, 2011**	1.000											
**Harbin, 2012**	0.923	1.000										
**Mudanjiang, 2011**	0.774	0.812	1.000									
**Mudanjiang, 2012**	0.751	0.799	0.891	1.000								
**Gongzhuling, 2011**	0.873	0.914	0.785	0.819	1.000							
**Gongzhuling, 2012**	0.800	0.747	0.663	0.633	0.790	1.000						
**Jinan, 2011**	0.741	0.793	0.759	0.825	0.790	0.553	1.000					
**Jinan, 2012**	0.768	0.882	0.801	0.871	0.844	0.557	0.874	1.000				
**Huaian, 2011**	0.873	0.902	0.837	0.868	0.880	0.693	0.877	0.889	1.000			
**Huaian, 2012**	0.876	0.906	0.832	0.863	0.878	0.710	0.881	0.895	0.978	1.000		
**Nanjing, 2011**	0.651	0.664	0.741	0.795	0.690	0.511	0.743	0.706	0.800	0.798	1.000	
**Nanjing, 2012**	0.626	0.629	0.708	0.767	0.686	0.476	0.696	0.704	0.776	0.779	0.938	1.000

**Figure 4:**
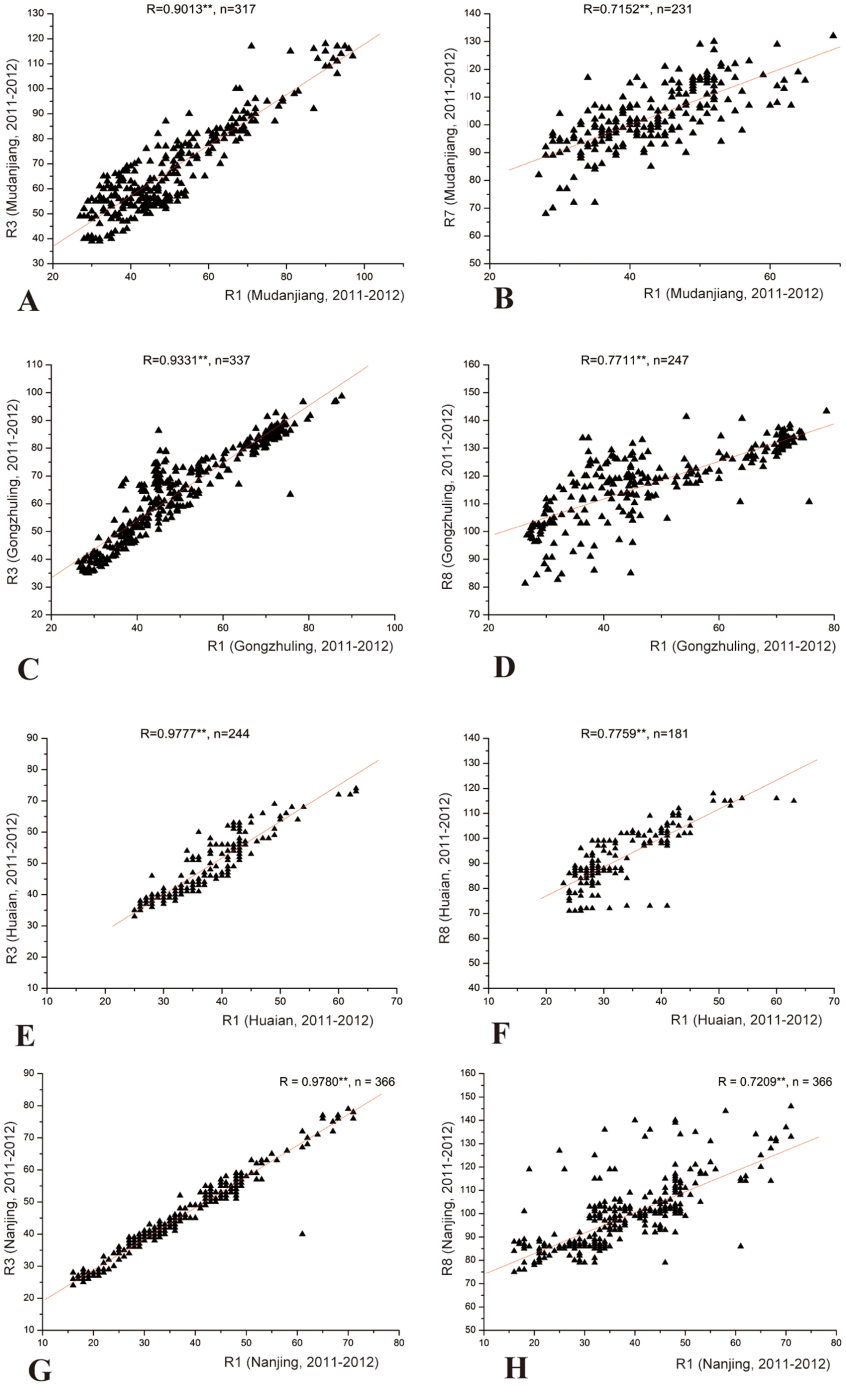
The correlation between R1 and R3, R7 or R8 at Nanjing, Huaian, Gongzhuling and Mudanjiang locations, average of the two years of 2011 and 2012. A: Correlation between R1 and R3 in Mudanjiang; B: Correlation between R1 and R7 in Mudanjiang. C: correlation between R1 and R3 in Gongzhuling; D: Correlation between R1 and R8 in Gongzhuling. E: Correlation between R1 and R3 in Huaian; F: Correlation between R1 and R8 in Huaian. G: Correlation between R1 and R3 in Nanjing; H: Correlation between R1 and R8 in Nanjing.

The number of days after emergence to the beginning of maturity (R7) and to fully maturity (R8) were measured in 2011 and 2012, statistical analysis showed a high correlation for R7 between 2011 and 2012 in Nanjing with a correlation coefficients of 0.941**. Generally, the correlation coefficient for R7 or R8 between different sites was lower than that for R1 (Table 2, Figure 3). Correlation coefficients between R1 and R3 were higher than that between R1 and R7 or R8 for all the sites (Figure 4).

**Table 2:** The correlation matrix for the maturity (R7 or R8) between all possible pairs at six locations in 2011 and 2012.

	R7_MDJ_11	R8_MDJ_11	R7_MDJ_12	R8_MDJ_12	R7_GZL_11	R8_GZL_11	R7_GZL_12	R8_GZL_12	R7_HA_11	R8_HA_11	R7_HA_12	R7_NJ_11	R7_NJ_12
**R7_MDJ_11**	1.000												
**R8_MDJ_11**	0.789	1.000											
**R7_MDJ_12**	0.554	0.545	1.000										
**R8_MDJ_12**	0.520	0.510	0.867	1.000									
**R7_GZL_11**	0.516	0.542	0.728	0.767	1.000								
**R8_GZL_11**	0.484	0.460	0.751	0.762	0.990	1.000							
**R7_GZL_12**	0.468	0.473	0.554	0.524	0.680	0.570	1.000						
**R8_GZL_12**	0.440	0.455	0.531	0.532	0.614	0.492	0.952	1.000					
**R7_HA_11**	0.525	0.605	0.639	0.666	0.629	0.700	0.635	0.596	1.000				
**R8_HA_11**	0.169	0.210	0.255	0.385	0.318	0.374	0.369	0.366	0.911	1.000			
**R7_HA_12**	0.133	0.109	0.358	0.332	0.313	0.232	0.506	0.500	0.766	0.740	1.000		
**R7_NJ_11**	0.169	0.215	0.291	0.269	0.277	0.318	0.196	0.189	0.623	0.670	0.668	1.000	
**R7_NJ_12**	0.163	0.157	0.271	0.287	0.249	0.271	0.252	0.258	0.683	0.691	0.723	0.941	1.000

**Note: MDJ, Mudanjiang; GZL, Gongzhuling; HA, Huaian; NJ, Nanjing; 11, 2011; 12, 2012.**

### 2. Allelic Variations at Four Major Alleles and Their Effects on Flowering Time and Maturity

We genotyped all the 180 cultivars (accessions) at the *E1*, *E2*, *E3* and *E4* genes in this study. Four allelic variations, *E1*, *e1-as*, *e1-fs*, *e1-nl*, were identified at the *E1* locus [19] (Figure 1A to C). According to multivariate analysis (Generalized Linear Models, Type III Sum of Squares in IBM SPSS), statistical significance level (P) for the effect of the *E1* allelic variation was within a range between 0.003 and 0.207 with an average of 0.0785 on flowering time (R1, Table S4) and a range between 0.00 and 0.435 with an average of 0.092 on maturity (R7 or R8, Table S5) in all geographic locations in 2011 and 2012.

Only two allelic variations, *E2* and *e2* were observed in this study (Figure 1D, [18]). The effect of *E2* allelic variations is reflected by significance levels (P) ranging from 0.005 to 0.657 with an average of 0.233 on flowering time (R1, Table S4), from 0.011 to 0.903 with an average of 0.449 on maturity (R7 or R8, Table S5).

Five allelic variations including three recessive alleles, *e3-fs*, *e3-tr*, *e3-Mo*, and two dominant alleles (*E3-Ha*, *E3-Mi*) were identified at the *E3* locus (Figure 1E to 1G) [17], [29], [36]. The statistical P values for the effects of *E3* allelic variations on flowering time were within a range from 0.02 to 0.422 with an average of 0.201 in 2011 and 2012 (Table S4). Also, effect of *E3* allelic variation on maturity (R7 or R8) was within a range from P = 0.02 to 0.964 with an average of 0.361 (Table S5).

For the *E4* allelic variations, *E4*, *e4-SORE1*, *e4-Kes*, and *e4-kam* were found among the 180 cultivars, the latter three alleles were recessive (Figure 1H to 1J) [16], [29], [30]. The significance levels (P value) for the effect of *E4* allelic variation were ranged from P = 0.029 to 0.869 with an average of 0.342 on flowering time (Table S4), and ranged from 0.022 to 0.238 with an average of 0.097 on maturity (R7 or R8, Table S5).

The percentages of recessive allele for *E1*, *E2*, *E3* and *E4*, were 38.34%, 84.45%, 36.33%, and 7.20%, respectively. Based on statistical analysis, interaction effects of E1*E2, E1*E3 and E2*E3 on flowering time (R1) were within statistical P values ranging from 0.056 to 0.892 (average 0.463), from 0.008 to 0.375 (average 0.109), and from 0.024 to 0.674 (average 0.336), respectively, among different geographic locations. The significant level (P) for the interaction effect for E1*E2*E3 was from 0.052 to 0.974 with an average of 0.591 (Table S4).

Similarly, interaction effects on maturity (R7 or R8) for E1*E2, E1*E3, E2*E3 were within ranges from 0.262 to 0.962 (average 0.628), from 0.003 to 0.873 (average 0.500), and from 0.013 to 0.659 (average 0.284), respectively, among different geographic locations.

### 3. Eight Genotypic Groups Were Classified

All the180 cultivars were classified into eight genotypic groups based on the combinations of allelic variations at the four major *E* loci, *E1*, *E2*, *E3*, and *E4* (Table 3).

**Table 3:** Genotypic groups were classified based on the allelic variations in *E1*, *E2*, *E3* and *E4* genes.

Genetic group	Genotype at *E1,E2L,E3, E4* genes	No of Cultivars	Geographic origin (frequency)
e1-nf	*e1-as,e2,e3-fs,e4-kes*	1	Heilongjiang(1)
	*e1-as,e2,e3-tr,e4-SORE1*	1	Heilongjiang(1)
	*e1-fs,e2,e3-tr,E4*	2	Japan(2)
	*e1-n1,e2,E3-Ha,e4-SORE1*	2	USA(2)
	*e1-n1,e2,E3-Mi,E4*	1	Japan(1)
	*e1-n1,e2,E3-Mi,e4-SORE1*	1	Japan(1)
	*e1-n1,e2,e3-tr,E4*	2	Heilongjiang(1), Canada(1)
	*e1-n1,e2,e3-tr,e4-SORE1*	3	Japan(1), Canada(1), Sweden(1)
e1-asE4	*e1-as,e2,e3-fs,E4*	5	Heilongjiang(1), Jilin(1), USA(3)
	*e1-as,e2,e3-tr,E4*	25	Heilongjiang(21), Jilin(3), Inner Mongolia(1)
E1-asE3E4	*e1-as,e2,E3-Ha,E4*	10	Jilin(3), Liaoning(1), Heilongjiang(1), China*(1), USA(4)
	*e1-as,e2,E3-Mi,E4*	7	Heilongjiang(4), Jilin(2), Liaoning(1)
e1-asE2(E3)E4	*e1-as,E2,e3-fs,E4*	1	Heilongjiang(1)
	*e1-as,E2,e3-Mo,E4*	2	Jilin(1), China* (1)
	*e1-as,E2,E3-Ha,E4*	5	Jilin(1), Shandong(1), Jiangsu(1), USA(2)
	*e1-as,E2,E3-Mi,E4*	1	Shandong(1)
E1	*E1,e2,e3-tr,e4-SORE1*	3	Japan(2), USA(1)
	*E1,e2,e3-tr,e4-kam*	1	Japan(1)
E1E4(E3)	*E1,e2,e3-fs,E4*	1	Jilin(1)
	*E1,e2,e3-tr,E4*	19	Heilongjiang(9), Liaoning(3), Jiangsu(1), Jilin(1), Japan(3), USA(2)
	*E1,e2,E3-Ha,e4-kes*	1	Jilin(1)
E1E3E4	*E1,e2,E3-Ha,E4*	20	Jilin(7), Jiangsu(3), Heilongjiang(2), Shandong(2), Xinjiang(1), Beijing(1), Henan(1), Anhui(1), USA(2)
	*E1,e2,E3-Mi,E4*	47	Jiangsu(20), Jilin (12), Liaoning(3), Heilongjiang(2), Beijing(2), China* (2), Gansu(1), Henan(1), Hubei(1), Xinjiang(1), Shandong(1), Japan(1),
E1E2E3E4	*E1,E2,E3-Ha,E4*	8	Jilin(2), Jiangsu(2), Heilongjiang(1), Liaoning(1), Shandong(1), USA(1)
	*E1,E2,E3-Mi,E4*	11	Jiangsu(6), Jilin(1), Heilongjiang(1), Beijing(1), Shanghai(1), Japan(1)
**Total**	**25**	**180**	

*No more detailed geographic information available.

#### (1) e1-nf Group

Cultivars in this group refer to as non-functional *E1* gene, consisting of three subtypes: *e1-fs* (frame-shift), *e1-nl* (null type), and *e1-as* (*e2*, *e3*, *e4*) (Tables 1, S1). First, cultivars Satamotowase and 9E carry *e1-fs* allele. Second, cultivars, Dongda 2, Toshidai 7910, Toyosuzu, and Yukihomare, are of *e1-nl*, lacking the *E1* gene and its proximate sequence. Third, the genotype of *e1-as,e2,e3,e4* is included in this group, considering that *e1-as* is partially functional and its expression is totally suppressed under long day condition under (*e2*) *e3e4* genetic background [19], e.g. Fiskeby V, a Sweden photoperiod insensitive cultivar (Table S1). The genotypes of several cultivars, e.g. Sakamotowase and Toyosuzu, are consistent with the result of Tsubokura et al. [29]. Geographically, three Chinese cultivars were coming from Heilongjiang, the most northern province of China. In addition, one, two, two, five accessions were from Sweden, the USA, Canada, and Japan, respectively.

**Figure 5:**
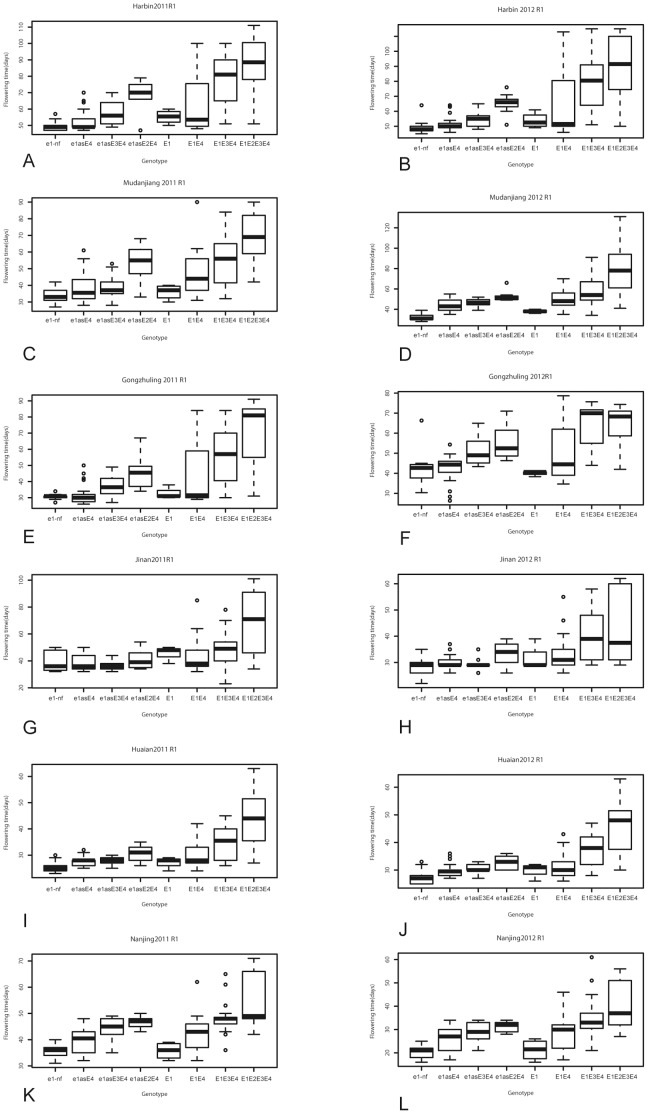
The phenotypic variations in R1 among different genotypic groups. The phenotypic segregation is shown in box-plot format. The interquartile region, median, and range are indicated by the box, the bold horizontal line, and the vertical line, respectively. The 12 panels (A to L) represented 6 experimental locations in 2011 and 2012, respectively. A: Harbin in 2011; B: Harbin in 2012; C: Mudanjiang in 2011; D: Mudanjiang in 2012; E: Gongzhuling in 2011; F: Gongzhuling in 2012; G: Jinan in 2011; H: Jinan in 2012; I: Huaian in 2011; J: Huaian in 2012; K: Nanjing in 2011; L: Nanjing in 2012.

Average R1 of this genotypic group was 34.35±10.01 days for all the six sites from 2011 to 2012. The longest R1 with an average of 49.54 days was observed in Harbin; and the shortest one was observed in Nanjing with an average of 20.31 days (Figure 5).

**Figure 6:**
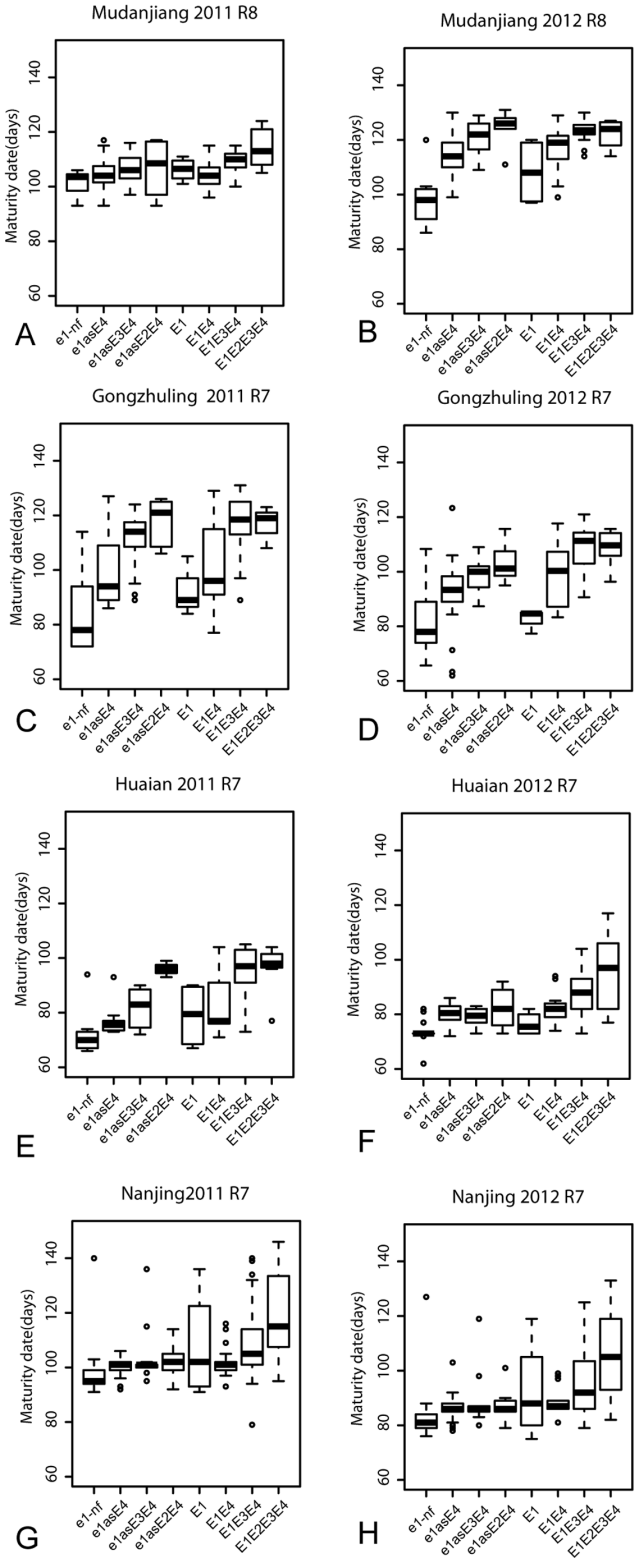
The phenotypic variations in R7 or R8 among different genotypic groups. The phenotypic segregation is shown in box-plot format. The interquartile region, median, and range are indicated by the box, the bold horizontal line, and the vertical line, respectively. A: R8 at Mudanjiang in 2011; B: R8 at Mudanjiang in 2012; C: R7 at Gongzhuling in 2011; D: R7 at Gongzhuling in 2012; E: R7 at Huaian in 2011; F: R7 at Huaian in 2012; G: R7 at Nanjing in 2011; H: R7 at Nanjing in 2012.

This group matured very early in all sites when compared with the other groups, and the ranges of R7 or R8 in the three northern sites were not significantly different from that in Southern sites (Figure 6). At the all sites, the averages of R7 and R8 were 85.83±14.58 days and 94.95±16.57 days, respectively.

#### (2) e1-asE4 Group

Cultivars or accessions of this group carry e*1-as* at the *E1* locus with dominant *E4* allele, while *e2* and *e3* are recessive (Tables 3, S1). Most cultivars in this group are being widely used in soybean cultivation in Heilongjiang, the major soybean production area in China, e.g. Heinong 44, Kengfeng 17, Hefeng 50, and Hefeng 55 (Table S1). Also some landraces from the northern regions of China are within this group. Average R1 of this genotypic group was 37.65±10.04 days for all six sites in 2011 and 2012 (Figure 6, Table S1). The longest R1 was observed in Harbin with an average of 52.31 days while the shortest R1 was observed in Nanjing with an average of 24.10 days.

#### (3) e1-asE3E4 group

Cultivars with genotypes of *e1-as*, recessive *e2*, and dominant *E3* and *E4* are classified into this group (Tables 3, S1). Cultivars in this group are mainly from Jilin and Liaoning provinces, both of which are located a few hundred kilometers south of Heilongjiang, in the northeast part of China. However, cultivars Hefeng 51 and Suinong 22 are widely used in Heilongjiang province (Table S1). Also cultivar Amsoy from the USA belongs to this group.

Average R1 of this genotypic group was 38.86±10.47 days for all the six sites in 2011 and 2012. The longest R1 was observed in Harbin with an average of 57.06 days while the shortest one was observed in Nanjing with an average of 27.88 days.

#### (4) e1-asE2(E3)E4 Group

Cultivars of this genotype are generally composed of two ecological types, *e1-asE2E4* and *e1-asE2E3E4* (Tables 3, S1). Although nine cultivars are classified into this genetic group, their origins are quite different; apart from three landraces and two foreign accessions, three cultivars, Gaofeng 1, Handou 5 and Sidou 11, were bred geographically in the Yellow River regions (Shandong, Hebei and the Northern region of Jiangsu province). The landraces, Moshidougong 503 and Xiaolimushidou are classified into the *e1-as-E2e3-MoE4* genotype (Table S1). The cultivar Moshidougong 503 has been used for linkage map construction and gene cloning of *E2* and *E3* [17], [18], [19], [37]. Cultivars with *e1-asE2E3E4* genotype were mainly coming from Shandong, Hebei or Northern Jiangsu, in downstream of the Yellow river.

Average R1 of this genotypic group was 46.13±14.29 days (Figure 5) for the six sites and in 2011 and 2012. The longest R1 of 58.56 days was observed in Harbin, and the shortest R1 with an average of 31.11 days was observed in Nanjing.

Average R7 and R8 of this genotypic group was 102.25±15.06 days for all six sites in 2011 and 2012. Average R8 of this genotypic group was 110.8±17.29 days for all six sites in 2011 and 2012 (Figure 6).

#### (5) E1 Group

Cultivars of this genetic group are characterized by dominant *E1* and recessive alleles of *e2*, *e3* and *e4* (Tables 3, S1). Apart from an American cultivar, three cultivars, Kariyutaka, 130 L and Iwahime, from Japan are of this genotype. No cultivar from China was classified into this group in this study.

Average R1 of this genotypic group was 36.64±10.56 days at the all six sites in 2011 and 2012 (Figure 5). The longest R1 was observed in Harbin with an average of 55.25 days, and the shortest one, an average of 21.25 days, was observed in Nanjing

Average R7 of this genotypic group were 92.33±15.46 days for all six sites and in 2011 and 2012 (Figure 6). While average R8 of this genotypic group was 101.87±14.25 days for all six sites in 2011 and 2012.

#### (6) E1E4 Group

This genetic group had dominant *E1* and *E4* alleles but with recessive *e2* and *e3* alleles (Tables 1, S1). Apart from some landraces and foreign cultivars, cultivars of this genotype are elite cultivars currently largely used in Heilongjiang, Jilin, and Liaoning Provinces.

Average R1 of this genotypic group was 43.76±16.96 days for all six sites in 2011 and 2012 (Figure 5). The longest R1 was observed in Harbin with an average of 65.81 days, while the shortest ones were observed in Nanjing with an average of 27.71 days.

Average R7 of this genotypic group was 98.22±13.23 days for all six sites in 2011 and 2012 (Figure 6). Average R8 of this genotypic group was 108.20±16.22 day for all six sites in 2011 and 2012 (Figure 6).

#### (7) E1E3E4 Group

Except for the recessive *e2* allele, *E1*, *E3* and *E4* alleles were dominant for this genetic group (Tables 3, S1). Of all 180 cultivars, 67 cultivars or accessions are classified into this group (Table 1), the largest one of eight groups classified in this study. The geographic distribution of this group is much diversified, from the northern Heilongjiang Province, to southern Jiangsu Province (the region along the Yangtze River).

Average R1 of this genotypic group was 53.21±18.98 days at all the six sites in 2011 and 2012 (Figure 5). The longest R1 was observed in Harbin with an average of 78.53 days, while the shortest one with an average of 31.54 days was observed in Huaian (Figure 5). At the maturity stage, average R7 and R8 of this genotypic group were 104.22±16.06 and 114.23±16.06 days at all the six sites in 2011 and 2012, respectively.

#### (8) E1E2E3E4 Group

Cultivars of this genetic group have all dominant *E1* to *E4* alleles, most of which are geographically from the southern areas, Jiangsu, Shanghai and Anhui Provinces (Tables 3, S1).

Average R1 of this genotypic group was 61.71±22.74 days for all the six sites in 2011 and 2012 (Figure 5). The longest R1 was observed in Harbin with an average of 90.83±40.16 days. The shortest one was observed in Nanjing with an average of 40.16 days (Figure 5). Average R7 of this genotypic group was 109.62±15.17 days for all six sites in 2011 and 2012. Average R8 of this genotypic group was 115.77±14.16 days for all six sites in 2011 and 2012 (Figure 6).

Some outliers (shown as dots in Figures 5 and 6) and representative cultivars showing contrasting R1 or R7 or R8 within the same genetic groups were selected for further phenotypic confirmation in 2013. The result showed that phenotypic performance in 2013 was consistent with that in 2011 and 2012 with high correlation coefficients (Table S6).

### 4. Comparison of Phenotypic Performance between Different Genetic Groups

We performed statistical comparisons of the time to flowering (R1) and maturity (R7 or R8) among different genetic groups.

#### (1) e1-nf *vs* E1

The genetic group of e1-nf carries either nonfunctional type of *e1* or partial functional *e1-as* coupled with recessive *e2*, *e3* and *e4* background. Flowering time (R1) or maturity (R7 or R8) of the e1-nf group were the earliest ones among all genetic groups. The E1 genetic group flowered a little later than the e1-nf group but not statistically different at all sites (Tables S7, S5). The E1 genetic group was only found in cultivars from Japan and the USA, including Kariyutaka, which was used for transformation of the *E1* gene for functional confirmation. Since recessive *e3e4* can suppress the expression of the *E1*or *e1-as* gene, E1 genotype carrying recessive *e2*, *e3* and *e4*, is phenotypically similar to the e1-nf. The subtle differences between the two groups might be detected if more cultivars of the *E1* type are used.

#### (2) e1-asE4 *vs* E1E4

The average difference of 14.31 days in flowering time (R1) between these two groups evaluated in Harbin 2012 reached a significant level of P<0.001 (Table S7). At the same site, the difference in R1 between the two groups in 2011 was also statistically significant (P<0.05). At another northern location Gongzhuling in 2011, the difference in R1 between the two groups was also significant at P<0.01 (Table S7). At Mudanjiang, another northern site, the difference in R1 was between 6.51 and 8.58 days (P<0.05) in the two years (Table S7). For R7 or R8 at all the six sites, the maximum difference between the two groups was up to 7.55 days, not statistically different (Table S8).

Assuming no other genes are involved in, the difference between two groups merely reflects the functional difference between *E1* and *e1-as* under a genetic background of *e2* and *e3*. The difference in R1 between the two groups appeared significantly only in the northern sites with longer day in this study.

#### (3) e1-asE3E4 *vs* E1E3E4

R1 observed at the north four sites between the two groups in 2011 and 2012 were all significantly different, while the difference at Huaian and Nanjing sites was not significant (Table S7). The difference in R7 between the two groups at Huaian in 2011 was significant (P<0.05); while both differences in R7 and R8 in Gongzhuling in 2012 were over 10 days, significantly different at P<0.01 (Table S8).

Since the *E3E4* background and long day condition can promote the expression of *E1* or *e1-as*, theoretically the functional difference between *E1* and *e1-as* can be clearly discerned under such genetic and environmental conditions.

#### (4) e1-asE2(E3)E4 *vs* E1E2E3E4

At all the sites the difference in R1 between two groups was from 8 to 28 days. In the most southern site Nanjing, the difference in R1 between the two groups in 2011 and 2012 was 8–9 days, although not significantly different (Table S7). For all other sites, the difference in R1 reached statistical significance (Table S7) in either 2011, or 2012, or both years.

At the most southern site, Nanjing, the difference in R7 between the two groups in 2011 and 2012 was 18–19 days, significantly at P<0.001 (Table S8). At another southern site, Huaian, the differences for R8 in 2011 and for R7 in 2012 between two groups were significant at P<0.01. The differences in R7 or R8 in the most northern sites, Mudanjiang and Gongzhuling did not occur at any statistical significant level possibly due to most cultivars in the *E1E2E3E4* genetic group not reaching the stages of R7 or R8 before frost. The comparisons between two genotypes indicated that the phenotypic difference in R1 between *e1-as* and *E1* under *E2E3E4* background were significantly larger than the other backgrounds.

#### (5) E1 *vs* E1E4

The differences in R1 at all locations in 2011 an2012 between the two groups did not reach statistical significance (Table S7). However, the significant difference (P<0.05) in R7 and R8 was not detected at any site in 2011 or 2012 between the two groups, except for R8 at Gongzhuling in 2012 at P<0.05 (Table S8). This result indicates that difference in functional effect of the *E1* gene under *e2e3E4* background rather than under the *e2e3e4* background is quantitative, but not significant.

#### (6) E1E4 *vs* E1E3E4

The differences in R1 between the two groups at Harbin site both in 2011 and 2012 were between 14.22 and 16.53 days (P<0.001) (Table S7). Also the differences at Gongzhuling in both 2011 and 2012 were 12.54 and 13.35 days, respectively, reaching statistical significance at P<0.001 (Table S7). At the all other sites, the differences between two groups were between 5 and 13 days, not significantly different (Table S7).

At maturity stage, the differences in R7 or R8 between two groups in Gongzhuling in 2011 and 2012 were significant at various level of P<0.05, P<0.01 or P<0.001. The differences in R7 in Huaian and Jinan in 2011 also reached significant level at P<0.05 and P<0.001, respectively (Table S8).

These results indicate that the function of the *E1* gene under *E3E4* background was more prominent than that under *e3E4* background; however, the difference between two backgrounds was reduced in the southern areas with shorter daylength at vegetative growing stage.

#### (7) E1E4 *vs* E1E2E3E4

Except for Jinan in 2012, the differences in R1 between two groups at all the locations in the two years were from 10 to 29 days, reaching statistical significant level at P<0.01, mostly at P<0.001 (Table S7).

At the later maturity stages, the differences in R7 or R8 between the two groups at the three northern locations were not significantly different. In contrast, at the three southern locations, the differences in R7 or R8 between two groups were larger, mostly at P<0.001 (Table S8). For example, the differences in R7 between two groups at Nanjing were from17.82 to 18.54 days (P<0.001, Table S8). In Mudanjiang and Gongzhuling, most cultivars in the *E1E2E3E4* genetic group could not mature before frost.

#### (8) E1E3E4 *vs* E1E2E3E4

Large differences of 15–21 days in R1 between the two groups were observed in Mudanjiang in 2011 and 2012 (P<0.001) (Table S7). In the most southern location Nanjing, no significant difference (6–7 days) in R1 between two groups was detected. In other sites, e.g. Harbin, Gongzhuling, Jinan, and Huaian, the differences in R1 between the two groups in 2011 and 2012 reached significant levels at P<0.05, P<0.01 or P<0.001 (Table S7).

At the later maturity stage, the difference in R7 and R8 at Huaian site in 2012 reached significance (P<0.01 or P<0.001) (Table S8). Similarly, in Nanjing, the differences in R7 in 2011 and 2012 reached significance level at P<0.001 level (Table S8).

The difference between two groups reflects the functions of the *E2* gene, which might not strongly be associated with photoperiod response [18].

### 5. Transcript Abundance of a Functional *E1* Allele Was Significantly Related to Flowering Time and Maturity

**Figure 7:**
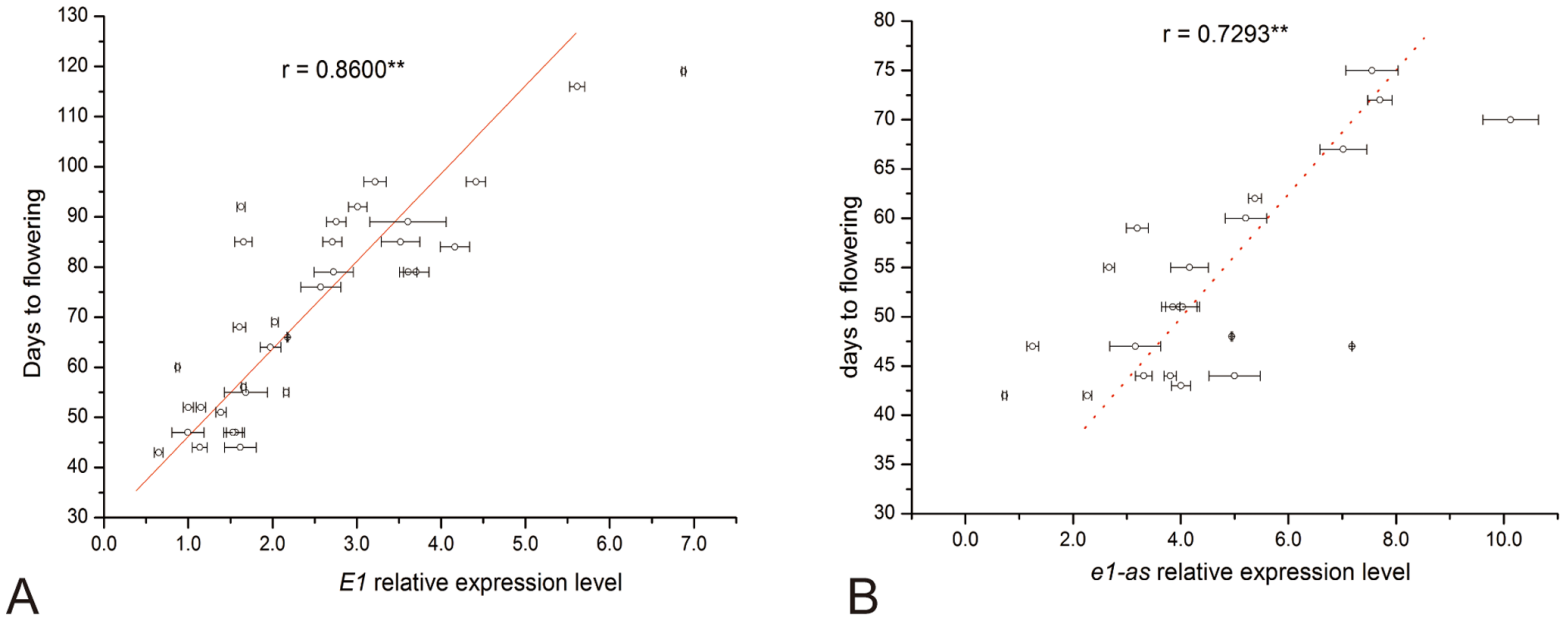
Correlation coefficients between the transcript abundance of the *E1* (A) or *e1-as* (B) genes and flowering time of cultivars grown at Harbin, in 2012.

Due to the prominent effect of the *E1* gene on flowering time, we analyzed the correlation between expression levels of functional *E1* gene and the flowering time. In the northern location of Harbin, we sampled the leaves of cultivars carrying the *E1* allele or *e1-as* allele within 2.5 to 3.0 hr after dawn at seedling stage (14 days after emergence) on May 20 (the day length was 16.10 h). The exact zeitgeber time (after dawn) for sampling was considered the first peak of a bimodal diurnal pattern for *E1* expression appears around 2–4 hr after dawn ([19], Abe et al, unpublished data). We analyzed the correlation between R1 and the expression levels of the *E1* (*e1-as*) gene, and a significant correlation was detected for the *E1* gene (r = 0.8600**, P<0.01) and for the *e1-as* gene (R = 0.7293**, P<0.01) (Figure 7). This result is consistent with that our previous work where the transcriptional level of the *E1* gene in transgenic soybean was significantly related to the flowering phenotype [19]. Also we can conclude that *e1-as* is also a functional allele at the *E1* locus, though its function in repressing flowering is not as strong as the *E1* gene.

## Discussion

Each soybean cultivar generally has a specific geographic or latitudinal distribution for its high yield. In soybean production, the northern cultivars with high photoperiod insensitivity flower very early when grown in the southern area, which limits biomass accumulation before flowering, and results a lower yield. On the other hand, the southern cultivars grown in the northern area might not be able to mature before frost. In Northern America, cultivars are classified into thirteen maturity groups based on the flowering time and maturity data. In China, researchers have also developed a system for classification of cultivars based on flowering time and maturity [38]. Some researchers have tried to synchronize different maturity group systems used in the USA and in China [39]. Understanding of the molecular basis or mechanisms involved in controlling maturing is very important for marker assisted selection (MAS) and for breeding by molecular design in the future.

### 1. The Magnitude of Effects of Four *E* Genes and Their Combinations on Flowering Time and Maturity

In this study, cultivars of the e1-nf group either having nonfunctional allele or putatively suppressed expression level of the *e1-as* gene displayed a very early flowering time phenotype, which confirmed that the *E1* is the most important locus for this trait. Similar to that of the *E1* gene, transcript abundance of *e1-as* gene was significantly correlated with flowering time, indicating *e1-as* is also a functional allele. Previous studies demonstrated that functional pathways of *E3* and *E4* are different but overlapping, at least partially through regulation of *E1* expression [19], [40]. Also, the effects of *E3* and *E4* revealed in this study are consistent with the results obtained in the previous genetic studies using Harosoy or Clark isogenic lines [20], [26]. The percentages of recessive alleles for the *E1*, *E2*, *E3* and *E4* loci were 38.34%, 84.45%, 36.33%, and 7.20%, respectively. This result indicates that dominant *E4* and recessive *e2* are the most common genetic makeups among Chinese cultivars, whereas the *E1* and *E3* loci have been undergone a high selection pressure in breeding in China.

### 2. Genes Other Than the Four Major *E1* Genes Exist

Among the known loci controlling flowering and maturity, *E5* to *E8*, and locus *J* have not been cloned. Recently, in some specific genotype backgrounds, some *Arabidopsis* flowering homologs were identified to be responsible for flowering time by QTL using mapping although further functional confirmation is needed (Watanabe et al, unpublished data). Since we have not sequenced the coding region or promoter region of *E1* to *E4* loci, there might be some structural or indel or SNP variations not yet detected [29]. As indicated in this study as well as the previous genetic study, some new genes are involved in control of flowering time, at least partially through regulation of the expression level of the *E1* (*e1-as*) gene.

### 3. Similar Phenotypic Performance for Flowering Time Can Be Achieved with Different Allelic Combination at the *E1* to *E4* loci

As revealed in this and previous studies [29], [30], there are a large number of allelic variations among the coding regions and promoter regions. Additionally, there are other known (e.g. *E5* to *E8*, *J*) or unknown genetic factors that are involved in the control of flowering time and maturity. More than one genetic groups identified in this study displayed similar or overlapping range of phenotypic performance among the cultivars. Theoretically, the improvement of a given trait can be achieved by various genetic combinations in breeding. On the other hand, if two cultivars displaying similar phenotype can have different genotypes, transgressive inheritance may occur in a cross between two such cultivars.

### 4. Future Prospective and Conclusion

Flowering time and maturity affect yield directly. Appropriate flowering time or maturity period are fundamentally required for soybean cultivars grown in different geographic regions. In Brazil, the introduce of the *J* locus, controlling the long juvenile trait, allowed the expansion of the soybean production area near north. Secondly, the *E* genes have effects on branching and growth habit. In 1930s, the breeding pioneer Jinling Wang already developed the most precocious cultivars, extending the frontier of soybean production zone further to the north by several hundred Kilometers in China [41]. Now we gradually understand that e1-nf genetic groups are approximately corresponding to cultivars of MG 000, MG 00, or MG 0 groups, since two cultivars from Canada and the cultivars from the most northern soybean production area, Heilongjiang, China belong to this e1-nf group.

According to a previous study, the *E1* gene also plays an important role in seed yield, plant height lodging and seed composition [26], since it can control *TFL1* expression [30]. On the other hand, the function of *E1* might also be related to branching trait, as indicated by a QTL mapping study [27]. In Canada, Cober and Morrison tried to use the *E1e3e4dt1* genotypic combination to elevate the position of the first pod [26].

One of the important goals for breeding is to improve yield of soybean cultivars. Considering that the flowering time and maturity are the simple but important traits affecting geographic adaption and yield [26], a full understanding of their precise regulation mechanism will enable us to breed cultivars with the appropriate flowering or maturity characters by design, possibly serve as a model for other more complicated traits e.g. yield and quality in soybean.

## Supporting Information

Table S1 **Generally information and phenotypic data of 180 accessions used in this study.**
(XLSX)

Table S2 **Primers used for genotyping in this study.**
(XLSX)

Table S3 **Flowering time (R1) and maturity (R7 or R8) data in different geographic locations in 2011 and 2012.**
(XLSX)

Table S4 **Multivariate analysis of allelic variations at** ***E1*** **,** ***E2*** **,** ***E3*** **, and** ***E4*** **and their combinations on flowering time (R1) using IBM SPSS Statistics 17.0 (www-01.ibm.com/software/analytics/spss) based on Generalized Linear Models (GML) and Type III Sum of Squares.**
(XLSX)

Table S5 **Multivariate analysis of allelic variations at** ***E1*** **,** ***E2*** **,** ***E3*** **, and** ***E4*** **and their combination on maturity (R7 or R8) using IBM SPSS Statistics 17.0 (www-01.ibm.com/software/analytics/spss) based on Generalized Linear Models and Type III Sum of Squares.**
(XLSX)

Table S6 **The correlation analysis of flowering time (R1) and R7 or R8 for about 50 cultivars (accessions) between 2013 and 2011/2012 at four experimental locations.**
(XLSX)

Table S7 **Two-way ANOVA analysis with Bonferroni posttests' test for flowering time (R1) of different genetic groups among different locations in 2011 and 2012 using GraphPad Prism version 5.00.**
(XLSX)

Table S8 **Two-way ANOVA analysis with Bonferroni posttests' test for maturity (R7 or R8) data of different genetic groups among different locations in 2011 and 2012 using GraphPad Prism version 5.00.**
(XLSX)
